# Humanity, Nature, and Survival in Yann Martel’s Life of Pi and Margaret Atwood’s Oryx and Crake: An Ecocritical Comparative Study

**DOI:** 10.12688/f1000research.183023.2

**Published:** 2026-09-02

**Authors:** Ayman Elhalafawy, Eman Abozied, Mohammed A. Adel, Ahmad M. Al Mahamed, Samir Khalifa

**Affiliations:** 1 Department of English Language and Literature at Faculty of Arts, Kafrelsheikh University, Kafr El-Shaikh, Egypt; 2 College of Humanities and Social Sciences, Department of Arabic Language and Literature, United Arab Emirates University, Al Ain, United Arab Emirates; 3 Department of Communication, College of Arts, Education and Social Sciences, Abu Dhabi University, Abu Dhabi, United Arab Emirates; 4 Medical Sciences and Preparatory Year Department, North Private College of Nursing, Arar, Saudi Arabia

**Keywords:** Ecocriticism; Humanity; Nature; Postcolonial Literature; Survival

## Abstract

Addressing the growing global urgency of environmental crises in contemporary Anglophone literature, this study examines the relationship between humanity, nature, and survival in Yann Martel’s *Life of Pi* and Margaret Atwood’s *Oryx and Crake* through an ecocritical Anthropocene framework. A significant gap remains in the absence of a comparative approach that integrates ecocriticism with postcolonial literary studies to analyze representations of humanity, nature, and survival under ecological pressure. This study explores how survival narratives construct ethical awareness, reshape human identity, and redefine human–nature relationships in the Anthropocene. Methodologically, it adopts a qualitative comparative approach combining close textual analysis, ecocriticism, and Anthropocene theory, aligning with interdisciplinary trends in English literary studies. The findings reveal a clear divergence: *Life of Pi* portrays nature as a formative and spiritually instructive force that fosters resilience and ethical growth, whereas *Oryx and Crake* depicts a degraded ecosystem reflecting ecological collapse, scientific misuse, and ethical failure. These representations of humanity, nature, and survival demonstrate how literature functions as a global ethical discourse that interrogates environmental responsibility. The study contributes to English literary studies by bridging literary theory with environmental humanities and recommends expanding comparative ecocritical research on Anthropocene literature.

## Introduction

This research explores how literature depicts the relationship between humans and nature and examines themes of survival, moral responsibility, and environmental awareness. The scope of the study is limited to the examination of two influential novels: Yann Martel’s *Life of Pi* (2001) and Margaret Atwood’s *Oryx and Crake* (2003). Both works depict humankind’s interaction with nature; however, they differ in how the authors present these ideas, both philosophically and through different time perspectives. Concisely, the novel by Yann Martel, *‘Life of Pi’,* portrays nature as a complicated, indomitable, and ultimately revitalizing entity of the spiritual side of man. The story is about Pi, a boy of Asian origin, who was stuck on a lifeboat in the middle of the Pacific Ocean along with a Bengal tiger, which he called Richard Parker. Along the way, nature becomes both his antagonist and his moral and spiritual guide. The endless sea, Richard Parker’s wild behavior, and Pi’s fight for survival illustrate nature’s role in the relationship between fear, faith, and instinct. Besides, the author is Canadian and is also known for writing *Beatrice and Virgil* (2010). In his works, Martel raises moral and existential issues through creative storytelling, and the book notably illustrates this by showing how nature becomes a human friend, sustenance, and ethical mediator. Conversely, in Margaret Atwood’s *Oryx and Crake*, we have a curtailed view of the coming times brought about by human greed, scientific experimentation, and pollution. In contrast to Martel’s celebratory depiction of nature, Atwood portrays the environment as vulnerable, subject to exploitation, and suffering from humanity’s constant meddling. Snowman, the main character, embodies the effects of environmental neglect and signifies survival as just one among the many challenges humanity faces. Atwood, a widely published Canadian author known for *The Handmaid’s Tale* (1985) and Cat’s Eye (1988), often weaves social, ethical, and environmental issues into her work. In *Oryx and Crake*, she warns of the repercussions of disregarding Earth’s limitations and the ethical responsibilities humans owe to nature. By comparing these two novels, this paper highlights how the portrayal of nature and humans’ fight for survival shifts across philosophical, temporal, and narrative contexts. Whereas *Life of Pi* presents nature as an ultimate source of guidance and enlightenment that teaches love and thereby moral and spiritual growth, *Oryx and Crake* depicts nature as being despoiled, controlled, and unrelentingly testing human ethical and physical strengths. This research seeks to answer the following questions: How do these novels depict humanity under environmental pressure? The authors redefine natural existence and life preservation through their actions. The solutions they offer demonstrate their understanding of current environmental issues affecting society in the twenty-first century. The study uses ecocritical theory to compare two novels, showing how fiction reveals human moral values and spiritual beliefs, and environmental aspects of contemporary life.

## Literature review

Scholarly attention to Yann Martel’s *Life of Pi* and Margaret Atwood’s *Oryx and Crake* has grown significantly in areas such as ecocriticism, the environmental humanities, and contemporary fiction studies. Comparative study directly contrasting the two works remains in its nascent stage, but existing research has already sufficiently illuminated their shared thematic concerns. These novels address environmental ethics, the interdependence of humans and nature, and survival in ecological crisis. Together, they provide a necessary theoretical basis for positioning both works within the literary response to environmental crisis and for understanding changing ideas of humanity in the twenty-first century.

Existing research shows broad agreement on the environmental and ethical concerns of both novels, particularly environmental ethics, the interdependence of humans and nature, and survival in ecological crisis. However, the studies differ in their emphasis on the representation of nature. According to Sarah Appleton (2019), Atwood’s dystopia warns of the consequences of a society that is reckless in its technological exploitation and neglectful of ecology. Her paper presents the novel as a potent critique of human-centered morality through its depiction of the natural world, which, as a victim of human interference, now fights for survival. Reid (2022) analyzes the portrayal of environmental disasters in today’s fiction. Reid points to a shift in modern literature, moving away from seeing nature as a moral guide toward seeing it as a victim of human excess. These studies emphasize environmental disasters, technological exploitation, human-centered morality, and nature as a victim of human excess.

By contrast, most literary studies focus on the spiritual and allegorical sides of nature. Daniel Bristow (2020) argues for applying a spiritual ecocritical lens to reading the novel and states that Martel depicts nature as the spiritual energy that drives the transformation of the main character’s identity. This paper supports interpreting the novel as an engagement with nature as a source of spiritual profundity, in stark opposition to Atwood’s dark ecological vision. Other interpretations read *Life of Pi* as a reflection of the survival-narrative trend in human-struggle literature. Monica Ghosh (2016) situates *Life of Pi* within modern literature that explores human frailty in harsh natural conditions. Ghosh (2021) argues that these stories serve as a vehicle for expressing changing views of nature, as writers use survival accounts to foreground the ecological and ethical aspects of human reliance on nonhuman life.

Taken together, these studies reveal different approaches to the representation of nature. While the research on *Oryx and Crake* emphasizes environmental disasters, technological exploitation, human-centered morality, and nature as a victim of human excess, the research on *Life of Pi* emphasizes spiritual profundity, human frailty, survival, and reliance on nonhuman life. Comparative study directly contrasting the two works remains in its nascent stage, despite these shared thematic concerns and different approaches to the relationship between humanity and nature.

Other literary studies offer critical frameworks for examining humanity’s fraught relationship with nature in *Oryx and Crake and Life of Pi.* Alhourani et al. (2025) frame identity and decolonization as narrative strategies in Martel’s reimagining of survival beyond colonial human–animal hierarchies. Moreover, Adel et al. (2025a) and Khalifa et al. (2025) emphasize the dissolution of human-centered binaries and relational subjectivity, offering Martel’s human–animal coexistence as a critique of human exceptionalism and a theoretical basis for reading Atwood’s genetically engineered species. Alenzi et al. (2026) further contextualize the present study by showing how the literary work questions social hierarchies and human conditions, which an ecocritical reading can support by linking class, humanity, and ethical responsibility within a broader Anthropocene and postcolonial environmental discourse. In addition, Elhalafawy et al. (2025) focus on crisis aesthetics by drawing conceptual parallels to the environmental disruption shaping Anthropocene fiction.

In addition, some papers indicate a growing ecocritical interest in *Life of Pi* and *Oryx and Crake*, particularly regarding environmental ethics. Recent studies confirm that environmental concern is no longer confined to literary or philosophical discourse but is increasingly being translated into legal and political frameworks aimed at protecting the environment and upholding human rights ( Khater et al., 2025a, 2025b; Khater, 2023). Critics of *Oryx and Crake* focus mainly on techno-capitalism, climate anxiety, and ecological collapse, interpreting Atwood’s novel as an environmental exploitation and post-truth culture ( Tasnim, 2025; Zainab & Aziz, 2025; Ateş, 2024). On the contrary, other ecocritical studies of *Life of Pi* highlight ecocentrism, spiritual ecology, and ethical relations between humans and non-human life, framing survival as an ethical and ecological transformation ( Shirlin & Selvaraj, 2025; Lison & Mani, 2023). These studies show valuable insights into humanity and nature, but lack a sustained comparative framework. Therefore, this paper addresses that gap by bringing *Crake* and *Life of Pi* into dialogue through an ecocritical lens, highlighting environmental ethics, an integrated idea of survival, and human responsibility in contemporary studies.

## Methodology

This study employs a qualitative, text-focused, and Eco-critically informed approach in the environmental humanities and comparative literary analysis. The method involves close reading to assess how each fiction depicts the bond between nature and humanity and how these depictions echo the changes in cultural and environmental issues. The study uses a conceptual and thematic framework to examine the role of narrative structure, figurative language, and characterization in responding to environmental pressure, spiritual crisis, and ecological collapse. This analysis focuses on ecocriticism, which examines the interaction between human experience and the nonhuman environment, making nature an active force that shapes narrative meaning and ethical issues. In this context, one of the texts presents the natural world as a factor that contributes to spiritual endurance and human survival. In contrast, the other portrays an environment that is ruined by technological greed and human supremacy. The paper also applies Anthropocene theory to examine the cultural, ethical, and environmental consequences of human intervention in planetary systems, especially in biotechnology, ecological imbalance, and environmental degradation. In this view, it is possible to discuss how modern fiction reflects the conflict between scientific development and ecological concerns. In addition, the study uses a comparative literature methodology to identify convergences and divergences in how the texts depict survival, humanity, and nature. Through close reading, the analysis examines symbolism, narrative, and thematic elements (such as depictions of animals, landscapes, and posthuman futures) and questions the ethical implications of these depictions. The comparative analysis is guided by the following research questions: How do *Life of Pi* and *Oryx and Crake* depict the relationship between humanity and nature? How do the two texts represent survival in relation to ecological crisis? What are the convergences and divergences between their ecological visions? How do the texts reflect the cultural, ethical, and environmental consequences of human intervention? By incorporating ecocriticism, Anthropocene studies, comparative analysis, and in-depth textual analysis, the study establishes a clear methodological framework for examining how literature not only echoes environmental crises but also shapes modern concepts of environmental responsibility and the human-nature relationship.

**Figure 1.**
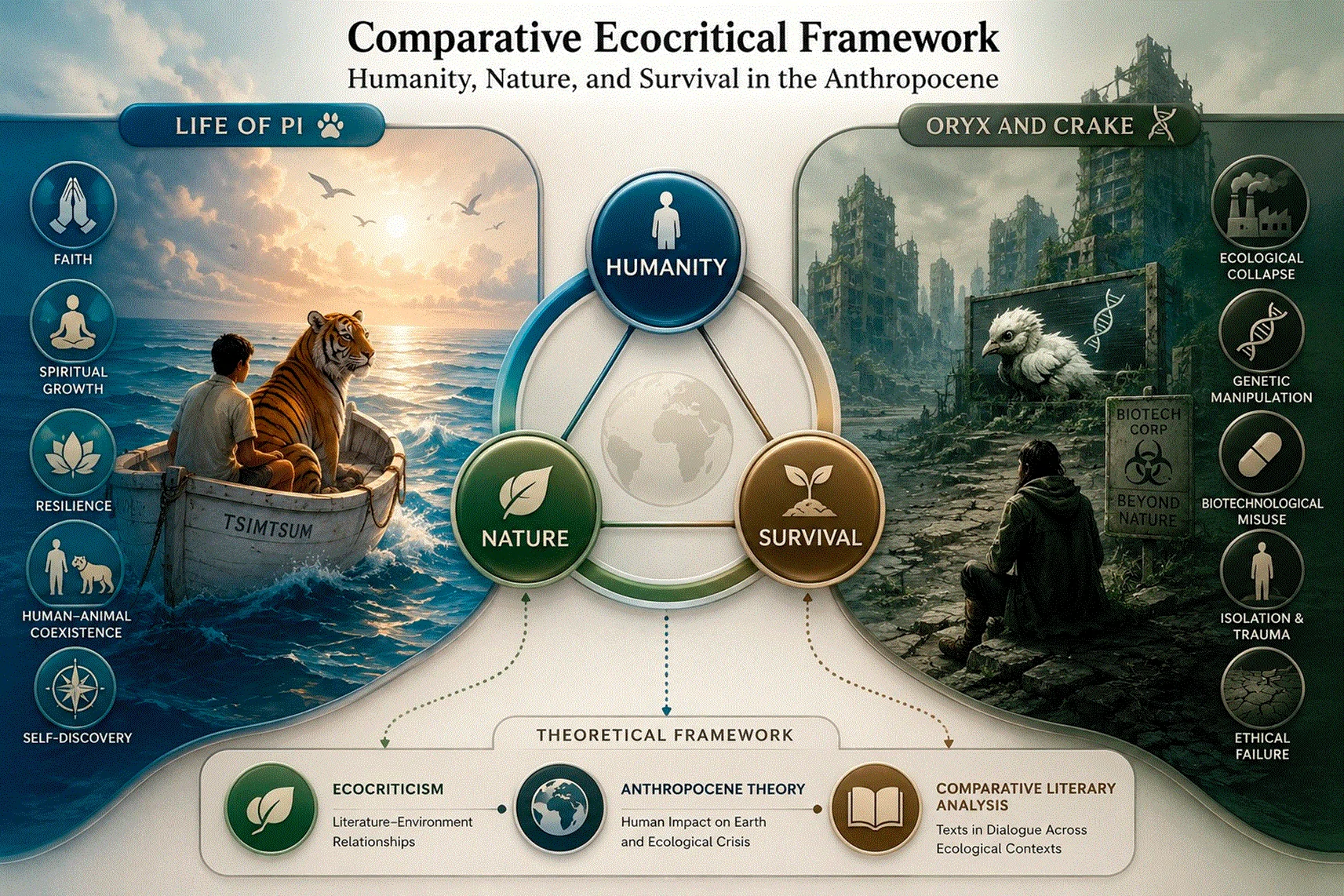
Comparative ecocritical framework of humanity, nature, and survival in Life of Pi and Oryx and Crake.

Figure 1 illustrates the study’s qualitative comparative framework by showing how *Life of Pi* and *Oryx and Crake* present contrasting views of humanity, nature, and survival: Martel depicts nature as spiritually formative, while Atwood portrays it as damaged by technological excess, ecological collapse, and ethical failure.

## Discussion

The environment is a crucial element in both *Life of Pi* by Yann Martel and *Oryx and Crake* by Margaret Atwood, as it shapes the human characters’ capacity to survive and their sense of self. There are numerous differences between the two books, including genre, tone, and environmental setting (the book by Martel is more of a spiritual survival, and the book by Atwood is a dystopian ecological collapse), but both of them have one thing in common: On their part, both books exhibit the similarity of identity as requiring some form of environmental pressure. Pi and Snowman, the protagonists of the two books, show that people change their psychological, moral, and emotional selves because of their surroundings.

In *Life of Pi*, nature is an active participant in shaping Pi’s character development rather than merely a backdrop. After the shipwreck, Pi had to face the huge and ruthless nature single-handedly. He thought to himself, “I was alone and orphaned, in the middle of the Pacific. I was all alone, and I had to make do with what I had” ( Martel, 2001, p. 131). The Pacific Ocean strips away the social, cultural, and familial roots Pi was holding on to, leaving him in a state of existential loneliness. As ecocritics Glotfelty & Fromm (1996) argue, nature is often depicted in literature as a “text of survival,” through which humans find their place and meaning in response to nonhuman forces. Pi’s ordeal is exactly like that; the sea is the means by which he reinvents his identity.

Martel presents nature’s two-sidedness through images that can be either glorious or frightening. The sea is termed “a boundless circle of blue, indifferent and absolute” ( Martel, 2001, p.102). Such apathy causes Pi to doubt his religion, morals, and self-concept. He even questions whether his strength is constructed against the sea, or in fact due to the sea. Each wave, storm, and time of hunger turns into a chapter of the book of survival. From an ecological point of view, Pi exemplifies Timothy Morton’s idea of “dark ecology,” a concept in which humans have to come to terms with the unpleasant side of their dependence on nature. Pi’s survival becomes a spiritual journey “under the instruction of nature,” whereby his weakness turns into self-realization.

Richard Parker makes the situation even more stressful for the environment. The tiger is not only a pet on the journey but also a sign of nature’s power in its most brutal form. Pi confesses, “Without Richard Parker, I would not be alive today to tell you my story” ( Martel, 2001, p.162). The tiger’s need for constant attention has driven the discipline, vigilance, and emotional control Pi could hardly have developed without the environmental threat Richard Parker represents. Instead of tearing Pi down, the tiger empowers his identity. The animal, in ecocritical terms, is a ‘nonhuman catalyst of human transformation’ that moves Pi to a higher level of awareness. His fear becomes his goal, his despair his strategy. Through this fight, Pi reveals an indomitable spirit that would have lain dormant in a secure environment.

On the other hand, *Oryx and Crake* portrays an environment that is not the result of nature’s grandeur but of human faults, scientific exploitation, and ecological collapse. Nevertheless, it has a deep impact on Snowman’s identity too. Atwood’s devastated world becomes her protagonist’s psychological mirror. Snowman refers to the post-apocalyptic world as one “where everything was gone: structures, people, sounds. Only the ruins remained” ( Atwood, 2003, p.45). His solitude, guilt, and disintegrated sense of self are in harmony with a landscape that has been broken too. Using the language of ecocriticism, Snowman is a subject born from the Anthropocene-induced changes of the planet.

While Pi’s environment supports his resilience, Snowman’s environment wears him down. He confesses, “I used to be Jimmy, but now I am Snowman—the last man, the leftover” ( Atwood, 2003, p.7). His change of name is a self-loss and environmental determinism metaphor. He is not reshaped through spiritual trial but trauma. The genetically modified animals, the lack of provisions, and the absence of human companionship push him into a never-ending cycle of terror and memory. The environment guides his every step, routine, and emotional reaction. The concept of trans-corporeality put forward by Stacy Alaimo suggests that the human body is closely related to the environment; therefore, Snowman’s mental fragmentation cannot be separated from the broken world he lives in.

While there is a stark contrast between Pi’s spiritual endurance and Snowman’s psychological decline, both books ultimately emphasize the idea that environmental pressures fundamentally change human identity. Pi is made by the environment; Snowman is destroyed by the environment. The ocean turns into a teacher for Pi, but the wasteland of the dystopia is the main cause of suffering for Snowman. However, both journeys show that people cannot figure out who they are without considering their ecological contexts. Thus, these two novels also support the first big finding of the study: environmental conditions are one of the most important determinants of human resilience and self-discovery, in other words, whether people will rise, adapt, collapse, or transform under the ecological pressure.

Both Yann Martel’s *Life of Pi* and Margaret Atwood’s *Oryx and Crake* detail the natural world in ways that are not only vivid but also very different, reflecting the philosophical, ethical, and narrative stance of each work. In the book *Life of Pi*, the natural world is represented as the setting and as the source of knowledge, as a motherly power which, in the course of the survival of the main character, contributes to the formation of his moral judgment and spiritual development. The immense Pacific Ocean challenges Pi to begin with, and he endures terror, death, and human endurance. He even says, “I must say a word about fear. It is life’s only true opponent. Only fear can defeat life” ( Martel, 2001, p.120). Nature is now the adversary and simultaneously the instructor: it is the irresolution that enables the hero to come to life again and to reflect morally. The ecocritics Glotfelty & Fromm cite that often in literature nature is a “‘text of survival,” in which the human identity and meaning are negotiated in relation to nonhuman forces. Moreover, Pi’s tribulations are a perfect example of that: every storm, hunger, or meeting with Richard Parker works as a school of self-discipline, awareness, and adaptation.

Martel’s narration is mainly about nature’s dual aspect: it is lovely at the same time as harmful. The ocean is called “a boundless circle of blue, indifferent and absolute” ( Martel, 2001, p.102). With this, however, Pi is reevaluating his faith, moral values, and self-perception. Every wave, storm, and moment of deprivation is a lesson, coining the term Timothy Morton’s “dark ecology,” and pointing towards human realization of their dependence on and vulnerability within the natural world ( Morton, 2016, p.18). Further, the tiger, Richard Parker, escalates the environmental pressure, as a “nonhuman catalyst of human transformation” ( Glotfelty & Fromm, 1996, p.45). Even Pi admits, “Without Richard Parker, I would not be alive today to tell you my story” ( Martel, 2001, p.162), thus exemplifying an animal that is both the challenge and the guide. As a result, Pi gains spiritual insight and grows spiritually strong through the ordeals, showing that self-exploration can depend on a nurturing nature.

On the other hand, Atwood’s *Oryx and Crake* depicts a frail, impoverished environment that reflects human mistakes, the exploitation of science, and moral neglect. Snowman is traveling through the world after the apocalypse, where the ecological disaster is the main obstacle to survival, and says, “Everything was gone: structures, people, sounds. Only the ruins remained” ( Atwood, 2003, p.45). Unlike Pi, whose environment teaches and strengthens him, Snowman’s world imposes trauma, fear, and isolation. Stacy Alaimo’s concept of trans-corporeality helps clarify this interaction by showing how the human body and environment are two aspects of one whole; Snowman’s mental and physical frailty reflects the ecosystem’s deterioration ( Alaimo, 2010, p.22). The idea of “slow violence” by Rob Nixon, which is used here to explain how the gradual environmental deterioration changes the human experience in a way that the psychological and social consequences are invisible at first, but after some time become very pronounced, further supports the idea of the novel ( Nixon, 2011, pp.2–3).

The post-human setting of Atwood’s novel is a sign that the ethical faculties have failed and that there will be moral repercussions. Snowman says, “I used to be Jimmy, but now I am Snowman—the last man, the leftover” ( Atwood, 2003, p.7), representing the loss of self, environmental determinism, and the erosion of identity as a result of the ecological disaster. Among the causes of the nature destruction in *The Oryx and Crake* universe - Genetically modified living things, scarcity of resources, and absence of human community - all lead to the realization that a dying nature will first of all threaten survival, ethical choices, and human psychology.

Greta Gaard’s work on ecofeminism strongly shows how environmental damage and social injustice are deeply interconnected ( Gaard, 1993, pp.45–47). Firstly, these novels articulate complementary yet contradictory perceptions of nature. *Life of Pi* shows how nature, as a mother, teacher, and challenger, can nurture and develop moral values. Atwood’s *Oryx and Crake* presents a fragile, damaged environment that warns of nature’s consequences and the consequences of neglect. Together, the two works convey that literature can view nature as a teacher and a reflection of the author’s ethical failure, underscoring the essential links between ecological conditions and human identity.

Both Yann Martel’s *Life of Pi* and Margaret Atwood’s *Oryx and Crake* depict fiction as a vital vehicle for imparting moral and existential values in relation to humanity’s interaction with nature. In *Life of Pi*, Martel develops a story in which survival goes hand in hand with ethical reflection, empathy, and even spiritual growth. Pi’s relation with Richard Parker is not just about the struggle for survival; it is a fight at the level of a moral code where responsibility, care, and living together are indispensable elements. As Pi thinks over the situation, “I had to tame him. It was not a question of dominance alone; it was a lesson in responsibility and coexistence” ( Martel, 2001, p.140). This instance is a perfect example of how fiction can invent a regulated world where ethical problems are solved, thus proving the interaction of human deed, moral decision, and environmental influence.

Glotfelty & Fromm claim that literature frequently depicts nature as a “text of survival” through which humans negotiate meaning and morality in reaction to nonhuman forces ( 1996, p.5). In Martel’s story, the sea and Richard Parker serve as didactic agents that shape Pi’s moral logic, self-control, and spiritual resilience. In addition, Martel’s allegorical mode and narrative creativity provoke the readers to confront the existential questions of human obligations to other species. The narrative is replete with examples of challenges to the survival of life, and at the same time, they serve as symbolic moral lessons.

Timothy Morton’s idea of “dark ecology” aligns with this interpretation: humans must face their reliance on the nonhuman world and, at the same time, acknowledge the vulnerability that comes with ecological relationships ( Morton, 2013, p.18). Hence, Pi’s narrative is an instance of the ability of literature to foster empathy, moral consciousness, and the capacity to withstand moral challenges via its creative depiction of nature.

Margaret Atwood’s *Oryx and Crake* uses dystopian fiction to show the effects of ethical neglect and environmental collapse. Snowman’s post-catastrophe world shows how human pride and ecological mismanagement create permanent danger to existence. The story here shows readers how destructive environmental exploitation will create an ethically depleted world. The character reports that “Everything that had been built was now in ruins. The arrogance of humans had changed the world” ( Atwood, 2003, p.58). The narrative centers on how human actions create permanent changes that affect nature, human values, and our understanding of existence. Rob Nixon’s concept of “slow violence” is at the core of this argument: the degradation of the environment is mostly a slow and hardly visible process; however, its moral and existential effects are long-lasting and deeply rooted ( Nixon, 2011, pp.3–4).

Snowman’s endurance of suffering shows how fiction lets people think about moral issues while connecting them to environmental disasters. His constant need to find moral solutions and understand his existence stems from three main influences: resource shortages, social isolation, and contact with genetically modified organisms. Stacy Alaimo’s concept of trans-corporeality explains how humans maintain their identity and moral values through interactions with their surrounding environment ( 2010). Atwood’s story shows that humans face direct and indirect consequences of environmental protection failures, which affect their survival, identity, and ethical standards. The two authors use different storytelling methods, yet their novels show that fiction can lead to moral and existential understanding. In *Life of Pi,* nature is portrayed as a positive educational force that helps people develop moral character and empathy. At the same time, *Oryx and Crake* depicts a destroyed world, which shows how human beings create environmental destruction through their failure to protect the natural world ( Adel et al., 2025b). Together, the two works show how imaginative literature can prompt readers to address ethical dilemmas, human responsibilities toward nature, and the ethical issues that arise during the Anthropocene. Together, the two works show how fiction can convey ethics and environmental awareness through storytelling.

## Findings

This paper analyzes Life of Pi by Yann Martel (2001) and Oryx and Crake by Margaret Atwood (2003) to understand the intricate connection between humans and nature, especially regarding resilience, self-discovery, and ethical contemplation. Through an ecocritical lens, the study shows that nature is not a passive literary backdrop but an active force that shapes human identity, morality, and survival. The role of nature in *Life of Pi* is both nurturing and educative. Because of its challenges, nature builds resilience, compassion, and spiritual awareness. *Oryx and Crake*, by contrast, presents a decayed, feeble world shaped by human exploitation and moral inadequacy, which heavily influence the protagonist’s fractured identity. Collectively, the writings highlight the inseparability of humanity and ecology. The comparative analysis shows three major conclusions: environmental situations play a significant role in human resilience and identity formation; the novels provide different images of nature as a nursery or a ravaged place; both works use fiction to share moral and existential lessons, and environmental responsibility is one of the main principles to stay alive. Overall, the study shows that literature is vital in developing environmental consciousness, moral contemplation, and a greater appreciation of the connection between humans and the natural world.

## Conclusion

This paper re-examined the dynamic interaction among humanity, nature, and survival in both Life of Pi by Yann Martel and Oryx and Crake by Margaret Atwood and established that environmental conditions are not just story settings, but determinants of human identity, ethical consciousness, and survival. The study contributes to the argument through a comparative ecocritical framework that shows how postcolonial fiction relates ecological pressure as a constitutive context in which moral responsibility and self-understanding are negotiated. In this respect, the study’s main contribution is its interpretive synthesis: comparing the ecological imaginaries that, in Life of Pi, sustain and morally educate, and in Oryx and Crake, appear as fragile, depleted objects, produces contrasting yet connected paradigms of survival. These results place literary survival stories in the context of wider discussions in postcolonial ecocriticism, anticipating the ability of fiction to find a voice to discuss global issues of environmental insecurity and human responsibility in the Anthropocene. Beyond its textual focus, the analysis highlights the study of literature’s contemporary relevance to environmental and ethical debates, positioning literature as a critical site where humanity’s ecological future is reflected. Future studies can apply this comparative ecocritical methodology to other postcolonial and global texts, especially those emerging from areas most affected by environmental precarity, further clarifying how literary forms respond to and restructure global ecological consciousness.

## Ethics approval

There are no studies involving human subjects in this article by any of the authors.

## Disclosure of AI use

The authors used OpenAI’s ChatGPT (GPT-5.6) premium only to generate the study’s visual abstract ( Figure 1). The tool was not used in writing, analysis, interpretation, or formulation of the manuscript’s findings. All AI-assisted output was reviewed and approved by the authors.

## Data Availability

Data sharing is not applicable to this article as no data were created or analysed in this research.
