# Supplementary material for: Humanity, Nature, and Survival in Yann Martel’s Life of Pi and Margaret Atwood’s Oryx and Crake: An Ecocritical Comparative Study
Source: F1000Res. 2026 Sep 2;15:1087. Originally published 2026 Jul 6. [Version 2] doi: 10.12688/f1000research.183023.2 (PMC13554829; doi:10.12688/f1000research.183023.2)
Supplement: Peer review history

# Reviewer report: Reviewer response for version 2

**DOI:** 10.5256/f1000research.209358.r517315

**Published:** 2026-09-15

**Authors:** Samia Mouas

**Affiliations:** 1 Department of English Language and Literature, Faculty of Letters and Foreign Languages, University of Batna 2, Batna, Algeria

I have no further comments and am happy to approve the article for publication.

Is the work clearly and accurately presented and does it cite the current literature?

Yes

If applicable, is the statistical analysis and its interpretation appropriate?

Not applicable

Are all the source data underlying the results available to ensure full reproducibility?

Yes

Is the study design appropriate and is the work technically sound?

Yes

Are the conclusions drawn adequately supported by the results?

Yes

Are sufficient details of methods and analysis provided to allow replication by others?

Partly

Reviewer Expertise:

Didactics of English as a Foreign Language (EFL), EFL pedagogy, teacher professional development, digital and blended learning, academic self-efficacy, higher-order thinking skills, and creativity in education.

I confirm that I have read this submission and believe that I have an appropriate level of expertise to confirm that it is of an acceptable scientific standard.

# Reviewer report: Reviewer response for version 2

**DOI:** 10.5256/f1000research.209358.r517314

**Published:** 2026-09-15

**Authors:** Abbas Abdelrady

**Affiliations:** 1 Department of English Language and Literature, College of Languages and Humanities, Qassim University,, Buraydah, Saudi Arabia

 The authors have adequately addressed the comments and concerns raised during the review process. The revised manuscript shows clear improvements, and the responses provided are satisfactory and appropriately justified. The modifications have enhanced the clarity and quality of the paper.

 I appreciate the authors' efforts in revising the manuscript and responding carefully to the reviewers' suggestions. I have no further major comments and recommend acceptance of the manuscript in its current form.

Is the work clearly and accurately presented and does it cite the current literature?

Yes

If applicable, is the statistical analysis and its interpretation appropriate?

Yes

Are all the source data underlying the results available to ensure full reproducibility?

Yes

Is the study design appropriate and is the work technically sound?

Yes

Are the conclusions drawn adequately supported by the results?

Yes

Are sufficient details of methods and analysis provided to allow replication by others?

Yes

Reviewer Expertise:

I have a PhD in English language and over 10 years of teaching experience. My area is applied linguistics.

I confirm that I have read this submission and believe that I have an appropriate level of expertise to confirm that it is of an acceptable scientific standard.

# Reviewer report: Reviewer response for version 1

**DOI:** 10.5256/f1000research.202023.r500558

**Published:** 2026-07-14

**Authors:** Samia Mouas

**Affiliations:** 1 Department of English Language and Literature, Faculty of Letters and Foreign Languages, University of Batna 2, Batna, Algeria

**Peer Review Report**

**Manuscript: “** *Humanity, Nature, and Survival in Yann Martel’s Life of Pi and Margaret Atwood’s Oryx and Crake: An Ecocritical Comparative Study”*

**Recommendation:** **Accepted with minor modifications**

 The manuscript offers an engaging comparative ecocritical reading of two significant works of contemporary fiction. The comparative framework is sound, the selection of primary texts is well justified by their shared environmental concerns, and the study makes a substantial contribution to the growing field of ecocritical and Anthropocene literary scholarship. The following points are raised as minor revisions that should be addressed before final publication.

**1. Literature Review**

 The literature review would benefit from a more critical synthesis rather than a sequential summary of individual studies. The authors are encouraged to reorganize this section around areas of scholarly agreement, points of theoretical disagreement, and unresolved debates in the field, rather than presenting sources one after another. This restructuring would sharpen the presentation of the study’s contribution without requiring new research.

**2. Self-Citation Balance**

 Approximately five of the manuscript’s thirty-five references are to the authors’ own prior work. While this proportion does not raise an ethical concern, the authors should consider incorporating a slightly broader range of external sources where appropriate, to strengthen the appearance of scholarly independence.

**3. Language and Prose Quality**

 A small number of sentences require light editing for clarity:

- “ *Dare to differ from Martel’s celebratory depiction of nature; Atwood characterizes the environment as a vulnerable entity*”: grammatically incorrect construction.
- “ *The work of the study is limited to the examination of two influential novels*”: awkward phrasing.
- “ *Through an ecocritical prism, reveals that nature is not the passive background*”: missing subject.
- More broadly, several paragraphs exceed a full page and should be divided for readability, and capitalization is inconsistent across terms such as Anthropocene, Ecocriticism, and Eco-critically.

Additionally, a few paragraphs exceed a full page and would benefit from being divided for readability, and capitalization should be made consistent across recurring terms such as Anthropocene and ecocriticism.

**4. Methodology**

- Add some explicit research questions or sub-questions to guide the comparative analysis in the methodology section.
- Consider providing a supplementary appendix listing the primary passages analyzed, the thematic categories used, and the analytical mapping between passages and categories, which would substantially improve transparency without requiring a change in the qualitative approach itself.
- Figure 1 is visually appealing but functions only as an illustration rather than an analytical tool; the manuscript never explains how the figure guided the analysis, and the image quality itself is noted as poor in places.

**5. Structural Organization**

 The Discussion section occasionally blends textual analysis with findings, making it slightly harder to distinguish supporting evidence from the conclusions drawn from it. A clearer separation between analytical evidence and interpretive synthesis in this section would improve the overall readability and argumentative clarity of the manuscript.

**Conclusion**

 These points are minor and editorial in nature and do not affect the validity or contribution of the study. Once addressed, the manuscript is suitable for publication.

**The Reviewer,**

 ……………………..

Is the work clearly and accurately presented and does it cite the current literature?

Yes

If applicable, is the statistical analysis and its interpretation appropriate?

Not applicable

Are all the source data underlying the results available to ensure full reproducibility?

Yes

Is the study design appropriate and is the work technically sound?

Yes

Are the conclusions drawn adequately supported by the results?

Yes

Are sufficient details of methods and analysis provided to allow replication by others?

Partly

Reviewer Expertise:

Didactics of English as a Foreign Language (EFL), EFL pedagogy, teacher professional development, digital and blended learning, academic self-efficacy, higher-order thinking skills, and creativity in education.

I confirm that I have read this submission and believe that I have an appropriate level of expertise to confirm that it is of an acceptable scientific standard.

# Response

**Published:** 2026-08-25

**Authors:** Mohammed Adel

**Affiliations:** United Arab Emirates University, Al Ain, Abu Dhabi, United Arab Emirates

**RESPONSE TO REVIEWER 2**

**Revised Version (Version 2)**

*Humanity, Nature, and Survival in Yann Martel’s Life of Pi and Margaret Atwood’s Oryx and Crake: An Ecocritical Comparative Study*

**Article**

 F1000Research 15:1087

**DOI**

 https://doi.org/10.12688/f1000research.183023.1

**Recommendation**

 Accepted with minor modifications

**Submitting author**

 ……….

 General response

 Dear Reviewer,

 We sincerely thank you for your positive evaluation of the manuscript and for recommending acceptance with minor modifications. We particularly appreciate your recognition of the sound comparative framework, the justification for selecting the two primary texts, and the study’s contribution to ecocritical and Anthropocene literary scholarship. We have carefully addressed each editorial recommendation, as detailed below. All textual changes are marked using Track Changes in the revised manuscript.

 Point-by-point responses

 Comment 1

**Reviewer comment: **Reorganize the Literature Review around scholarly agreement, theoretical disagreement, and unresolved debates rather than presenting a sequential summary of individual studies.

**Response: **We agree that a critical synthesis strengthens the articulation of the research gap and the study’s contribution.

**Changes made: **The Literature Review has been reorganized thematically. It now first identifies areas of agreement concerning environmental ethics, human dependence on nonhuman life, and survival under ecological crisis. It then distinguishes contrasting scholarly emphases: technological exploitation, ecological collapse, and posthuman ethics in Oryx and Crake versus spiritual ecology, allegory, and human–animal interdependence in Life of Pi. The final part identifies the unresolved debate and research gap: the absence of a sustained comparative account of how these opposing ecological imaginaries produce different models of survival and environmental responsibility.

**Location in the revised manuscript: **Literature Review, particularly the opening synthesis, the contrasting strands on the two novels, and the concluding research-gap paragraph.

 3. Language and Prose Quality

 Comment 2a

**Reviewer comment: **Revise the grammatically incorrect construction: ‘Dare to differ from Martel’s celebratory depiction of nature; Atwood characterizes the environment as a vulnerable entity.’

**Response: **We agree and have corrected the sentence.

**Changes made: **The sentence has been rewritten as: ‘In contrast to Martel’s celebratory depiction of nature, Atwood portrays the environment as vulnerable, subject to exploitation, and damaged by persistent human intervention.’

**Location in the revised manuscript: **Introduction, paragraph contrasting the ecological visions of the two novels.

 Comment 3b

**Reviewer comment: **Revise the awkward phrase: ‘The work of the study is limited to the examination of two influential novels.’

**Response: **We agree and have replaced the awkward construction with a direct statement of scope.

**Changes made: **The revised sentence reads: ‘The scope of the study is limited to two influential novels: Yann Martel’s Life of Pi and Margaret Atwood’s Oryx and Crake.’

**Location in the revised manuscript: **Introduction, opening paragraph.

 Comment 3c

**Reviewer comment: **Correct the sentence fragment: ‘Through an ecocritical prism, reveals that nature is not the passive background.’

**Response: **We agree and have supplied the missing grammatical subject.

**Changes made: **The revised sentence reads: ‘Through an ecocritical lens, the study reveals that nature is not a passive literary backdrop but an active force shaping human identity, morality, and survival.’

**Location in the revised manuscript: **Findings, opening paragraph.

 Comment 3d

**Reviewer comment: **Divide paragraphs that exceed a full page and standardize capitalization in terms such as Anthropocene, Ecocriticism, and Eco-critically.

**Response: **We agree and have completed a broader language and consistency edit.

**Changes made: **Long paragraphs have been divided according to claim, evidence, theoretical interpretation, and comparative implication. Capitalization has been standardized: ‘Anthropocene’ is capitalized when referring to the geological and theoretical concept, while ‘ecocriticism,’ ‘ecocritical,’ and ‘ecocritically’ are written in lowercase except at the beginning of a sentence.

**Location in the revised manuscript: **Throughout the manuscript, especially the Literature Review, Methodology, Discussion, and Findings.

 4. Methodology

 Comment 4a

**Reviewer comment: **Add explicit research questions or sub-questions to guide the comparative analysis in the Methodology section.

**Response: **We agree and have added explicit research questions to align the procedure with the analytical aims.

**Changes made: **The Methodology now specifies four guiding questions addressing: (1) representations of humanity–nature relations; (2) survival under ecological crisis; (3) convergences and divergences between the novels’ ecological visions; and (4) the cultural, ethical, and environmental consequences of human intervention.

**Location in the revised manuscript: **Methodology, research-questions paragraph.

 Comment 4b

**Reviewer comment: **Consider providing a supplementary appendix listing the passages analyzed, thematic categories, and the mapping between passages and categories.

**Response: **We appreciate this valuable suggestion. Because the recommendation is optional and the article is a qualitative literary study based on publicly available primary texts, we have strengthened transparency within the manuscript rather than adding a separate supplementary appendix at this stage.

**Changes made: **The Methodology now explains the criteria for selecting passages, the thematic coding process, the categories used, the cross-text comparison, and the procedures used to validate interpretations. The revised methodological figure also maps the relationship between passage selection, coding, thematic grouping, theoretical interpretation, comparison, and synthesis. We would be pleased to provide an appendix if the Editorial Team considers it necessary.

**Location in the revised manuscript: **Methodology, analytical-procedure and validation paragraphs; revised Figure 1.

 Comment 4c

**Reviewer comment: **Figure 1 is illustrative rather than analytical; explain how it guided the analysis and improve its image quality.

**Response: **We agree and have revised both the function and presentation of Figure 1.

**Changes made: **Figure 1 has been redesigned as a methodological workflow rather than a content illustration. It now maps corpus selection, purposive passage selection, initial coding, thematic grouping, ecocritical and Anthropocene interpretation, cross-text comparison, validation, and interpretive synthesis. The accompanying paragraph explains how these stages guided the analysis. A new high-resolution version is supplied as a separate figure file for production.

**Location in the revised manuscript: **Methodology, Figure 1 and its accompanying explanatory paragraph; separate high-resolution figure file.

 5. Structural Organization

 Comment 5

**Reviewer comment: **Clarify the distinction between textual analysis and findings in the Discussion section.

**Response: **We agree that clearer functional separation improves the manuscript’s argumentative progression.

**Changes made: **The Discussion has been revised to focus on analytical evidence: specific scenes, images, metaphors, narrative structures, and their theory-guided interpretation. Comparative synthesis is placed at the end of the relevant analytical units, while the Findings section consolidates the principal conclusions without reproducing the supporting textual analysis. Repetitive statements have been removed or relocated.

**Location in the revised manuscript: **Discussion throughout and the opening and synthesis paragraphs of the Findings section.

 Closing statement

 We again thank the reviewer for the careful and constructive recommendations. These revisions have improved the critical synthesis of the literature, citation balance, linguistic clarity, methodological transparency, figure quality, and structural distinction between analysis and findings. We hope that the revised manuscript now fully satisfies the requested minor modifications.

 Sincerely,

# Reviewer report: Reviewer response for version 1

**DOI:** 10.5256/f1000research.202023.r500555

**Published:** 2026-07-13

**Authors:** Abbas Abdelrady

**Affiliations:** 1 Department of English Language and Literature, College of Languages and Humanities, Qassim University,, Buraydah, Saudi Arabia

**Peer Review Report**

**Strengths **

1. Topic has relevance to ecocriticism scholarship and Anthropocene literary studies in general.
2. Comparative study of Life of Pi and Oryx and Crake yields interesting contrast; concept is strong .
3. Authors demonstrate knowledge of current scholarship. Successfully weave several theoretical frameworks into analysis .
4. Textual readings are insightful at times, particularly noting the portrayal of nature as spiritually formative in Life of Pi versus degraded/source of fear/pollution in Oryx and Crake.

**Weaknesses **

1.
  1. Over-conceptualized. Methodology section lists numerous theoretical frameworks without narrowing focus on one or two lenses ([ECO]criticism, Anthropocene Theory, Comparative Literature, Postcolonial Theory, Symbolism Analysis, Narrative Analysis, Crisis Aesthetics etc.)
  2. Authors fail to articulate how close readings were performed (methodology for selection, coding, thematic extraction, etc.)
  3. Rationale for including two novels in comparative study not explained.
  4. Very little textual evidence; authors retell novels rather than analyze
  5. Some theoretical concepts seem tenuous or incorrect (? what is meant by “dark ecology”?)
  6. Non ecocriticism citations littered throughout literature review section (tourism, humanitarian assistance, Arabic dubbed cartoons)
  7. Intro has unnecessary plot summary, lit review lacks focus, discussion largely repeats information from previous sections. , ,
  8. Claim in conclusion (" bridges literary theory built around landscape and environment with emergent scholarship in environmental humanities worldwide to"…) far too broad
  9. References lack page #'s, contains irrelevant self citations, etc.

**Suggested Corrections**

 1. Methodology

1.
  1. Trim down number of theoretical frameworks. Choose one main lens ([ECO]criticism) and one supporting lens (Anthropocene theory) through which to view texts.
  2. Add specific description of procedure used when conducting analysis including:
  3. How were passages selected?
  4. How were themes/codes extracted?
  5. How were authors’ interpretations validated?
  6. Provide brief but direct explanation of why Life of Pi was compared to Oryx and Crake (ie: because they portray opposing ecological imaginaries)
  7. Figure 1 should map your methodological steps, not describe the content of the novels.

2. Analysis

1.
  1. Minimize plot summary and replace with close reading of specific scenes, images/metaphors, symbols, plot structures, etc.
  2. Ground theoretical concepts in textual evidence and apply them directly (ie. illustrate or provide examples of how “slow violence” manifests in the text of Oryx and Crake )
  3. Build stronger synthesis by articulating how reading these two novels together produced new insights. (What does it tell us that we can only see when these novels are read in conversation? Shared anxieties? Different ecological ethics?)

3. Literature Review

1.
  1. Delete all citations not related to ecocriticism. (tourism, humanitarian assistance, cartoons)
  2. Organize lit review into three concise strands:
  3. Explain connection between postcolonial theory and ecocriticism if you want this theory to stay in your framework.

4. Writing & Structure

1.
  1. Authors should pare down introduction. Focus on research gap rather than summary of both novels’ plots.
  2. Discussion section should provide new information. It should not be repeating information found in introduction and lit review sections.
  3. Paragraphs are very long and should be broken up into more digestible pieces.

5. Conclusions

 Narrow your conclusion down to what this study specifically adds. Don't make far-reaching claims about how your study " connects" literary theory to ethics worldwide.

 6. References

 Delete all unnecessary citations. Delete all self citations not used in essay.

Is the work clearly and accurately presented and does it cite the current literature?

Yes

If applicable, is the statistical analysis and its interpretation appropriate?

Yes

Are all the source data underlying the results available to ensure full reproducibility?

Yes

Is the study design appropriate and is the work technically sound?

Yes

Are the conclusions drawn adequately supported by the results?

Yes

Are sufficient details of methods and analysis provided to allow replication by others?

Yes

Reviewer Expertise:

I have a PhD in English language and over 10 years of teaching experience.

I confirm that I have read this submission and believe that I have an appropriate level of expertise to confirm that it is of an acceptable scientific standard.

# Response

**Published:** 2026-08-25

**Authors:** Mohammed Adel

**Affiliations:** United Arab Emirates University, Al Ain, Abu Dhabi, United Arab Emirates

**RESPONSE TO REVIEWER**

 General response

 Dear Reviewer,

 We sincerely thank you for your careful reading of our manuscript and for your constructive and detailed recommendations. Your comments helped us improve the study’s methodological transparency, analytical depth, theoretical focus, organization, and overall clarity. We have revised the manuscript extensively and have addressed each point below. All changes are visible through Track Changes in the revised manuscript.

**Response to the strengths identified**

 We appreciate the reviewer’s positive assessment of the study’s relevance to ecocriticism and Anthropocene literary studies, the value of comparing Life of Pi and Oryx and Crake, the engagement with recent scholarship, and the distinction between nature as spiritually formative in Martel’s novel and as degraded and threatening in Atwood’s novel. We have retained these strengths while revising the manuscript to sharpen its central argument and comparative contribution.

**A. Responses to the identified weaknesses**

 Comment A1

**Reviewer comment: **The study is over-conceptualized, and the Methodology section lists too many theoretical frameworks without narrowing the focus.

**Response: **We agree. The theoretical architecture has been streamlined so that ecocriticism functions as the principal analytical lens and Anthropocene theory as the supporting lens. Other approaches are no longer presented as independent, co-equal frameworks.

**Changes made: **The Methodology section now distinguishes the study’s two theoretical lenses from its analytical procedures. Comparative reading, close reading, attention to narrative structure, and thematic analysis are presented as procedures rather than additional theories.

**Location in the revised manuscript: **Methodology section, opening and theoretical-framework paragraphs.

 Comment A2

**Reviewer comment: **The authors do not explain how the close readings were performed, including passage selection, coding, and thematic extraction.

**Response: **We agree that the earlier version did not provide sufficient procedural detail. We have revised the Methodology section to make the analytical process more transparent and reproducible.

**Changes made: **The revised methodology explains that passages were purposively selected when they represented environmental pressure, human–nonhuman relations, survival, ecological degradation, biotechnology, or ethical responsibility. The selected passages were coded, grouped into recurring themes, compared across the two novels, and checked against the study’s research questions and theoretical definitions.

**Location in the revised manuscript: **Methodology section, analytical-procedure paragraph.

 Comment A3

**Reviewer comment: **The rationale for including the two novels in the comparative study is not explained.

**Response: **We agree and have made the selection rationale explicit.

**Changes made: **The revised manuscript explains that the two novels were selected because they offer opposing but complementary ecological imaginaries: Life of Pi portrays nature as dangerous yet spiritually formative, whereas Oryx and Crake portrays an environment damaged by technological excess and anthropogenic intervention. Their comparison therefore clarifies contrasting models of survival and environmental ethics.

**Location in the revised manuscript: **Introduction, research-gap paragraph; Methodology, corpus-selection rationale.

 Comment A4

**Reviewer comment: **There is very little textual evidence, and the manuscript retells the novels rather than analyzing them.

**Response: **We agree. Plot summary has been reduced and the analysis has been strengthened through closer engagement with textual details.

**Changes made: **The Discussion now gives greater attention to scenes, images, metaphors, naming, landscape, the lifeboat and ocean, Richard Parker, Snowman’s ruined environment, genetically modified beings, scarcity, and narrative structures. Quotations are followed by interpretation linking textual form to ecological meaning.

**Location in the revised manuscript: **Discussion section, especially the analyses of the Pacific Ocean, Richard Parker, Snowman’s renaming, ecological ruins, and genetically modified life.

 Comment A5

**Reviewer comment: **Some theoretical concepts appear tenuous or incorrect, including the use of ‘dark ecology.’

**Response: **We appreciate this important observation. We have clarified the concept and restricted its use to passages that directly support it.

**Changes made: **Dark ecology is now defined as an encounter with the disturbing, uncomfortable, and entangled character of ecological coexistence. Its application is tied specifically to Pi’s simultaneous dependence on and vulnerability to the sea and Richard Parker. The concept is no longer used as a general label for every environmental difficulty.

**Location in the revised manuscript: **Discussion section, paragraph interpreting the ocean’s indifference and Pi’s dependence on nonhuman life.

 Comment A6

**Reviewer comment: **The Literature Review contains citations unrelated to ecocriticism, including work on tourism, humanitarian assistance, and Arabic-dubbed cartoons.

**Response: **We agree. The Literature Review has been refocused on scholarship that directly supports the study’s ecocritical and Anthropocene concerns.

**Changes made: **Tangential citations have been removed from the literature-review argument, and the review now prioritizes scholarship on environmental ethics, ecological collapse, survival narratives, spiritual ecology, human–nonhuman relations, and Anthropocene fiction.

**Location in the revised manuscript: **Literature Review and corresponding reference-list revisions.

 Comment A7

**Reviewer comment: **The Introduction contains unnecessary plot summary, the Literature Review lacks focus, and the Discussion repeats material from earlier sections.

**Response: **We agree and have revised all three sections for clearer functional differentiation.

**Changes made: **The Introduction now foregrounds the problem, gap, aims, questions, and comparative rationale; the Literature Review is organized around relevant scholarly strands; and the Discussion is reserved for textual interpretation and comparative synthesis rather than repeating background material.

**Location in the revised manuscript: **Introduction, Literature Review, and Discussion throughout.

 Comment A8

**Reviewer comment: **The claim in the Conclusion that the study bridges environmental literary theory with environmental humanities worldwide is too broad.

**Response: **We agree. The conclusion has been narrowed to avoid a universal or disproportionate claim.

**Changes made: **The revised conclusion identifies the study’s specific contribution as an interpretive synthesis of two contrasting ecological imaginaries and two connected paradigms of survival. Broader claims about ethics worldwide have been removed or qualified.

**Location in the revised manuscript: **Conclusion, contribution paragraph.

 Comment A9

**Reviewer comment: **The references lack page numbers and include irrelevant citations.

**Response: **We agree that citations must be precise and directly relevant.

**Changes made: **Page numbers have been added where specific passages or concepts are quoted or closely discussed. Unnecessary citations and citations that do not contribute directly to the argument have been removed from the text and reference list.

**Location in the revised manuscript: **Discussion citations and References section.

**B. Responses to the suggested corrections**

 1. Methodology

 Comment M1

**Reviewer comment: **Choose one main lens (ecocriticism) and one supporting lens (Anthropocene theory).

**Response: **We agree and have adopted this recommendation.

**Changes made: **Ecocriticism is now the principal lens, with Anthropocene theory serving as a supporting framework for human intervention, biotechnology, ecological imbalance, and environmental degradation.

**Location in the revised manuscript: **Methodology, theoretical-framework paragraphs.

 Comment M2

**Reviewer comment: **Add a specific description of the procedure used to conduct the analysis.

**Response: **We agree and have added a sequential account of the qualitative procedure.

**Changes made: **The method now moves from corpus selection and purposive passage selection to initial coding, thematic clustering, theory-guided interpretation, cross-text comparison, and interpretive validation.

**Location in the revised manuscript: **Methodology, analytical-procedure paragraph; revised Figure 1.

 Comment M3

**Reviewer comment: **Explain how passages were selected.

**Response: **We agree.

**Changes made: **The revised manuscript states that passages were selected when they contained sustained representations of environmental pressure, survival, animals or other nonhuman life, altered landscapes, ecological collapse, biotechnology, or environmental responsibility.

**Location in the revised manuscript: **Methodology, passage-selection description.

 Comment M4

**Reviewer comment: **Explain how themes and codes were extracted.

**Response: **We agree.

**Changes made: **The revised procedure describes initial descriptive coding of recurrent textual features, followed by grouping the codes into the study’s central themes: ecological pressure and identity, contrasting ecological imaginaries, survival and ethical responsibility, and human–nonhuman interdependence.

**Location in the revised manuscript: **Methodology, coding and thematic-extraction description.

 Comment M5

**Reviewer comment: **Explain how the authors’ interpretations were validated.

**Response: **We agree.

**Changes made: **Interpretations were checked through repeated reading, comparison with the full narrative context, cross-text comparison, consistency with the stated theoretical definitions, and collaborative review among the authors. Claims not supported by textual or theoretical evidence were revised or removed.

**Location in the revised manuscript: **Methodology, interpretive-validation description.

 Comment M6

**Reviewer comment: **Provide a direct explanation of why Life of Pi was compared with Oryx and Crake.

**Response: **We agree.

**Changes made: **The revised study states that the novels were paired because they stage opposing ecological imaginaries while sharing central concerns with survival, human vulnerability, nonhuman agency, and environmental responsibility.

**Location in the revised manuscript: **Introduction, comparative rationale; Methodology, corpus selection.

 Comment M7

**Reviewer comment: **Figure 1 should map the methodological steps rather than describe the content of the novels.

**Response: **We agree and have redesigned the figure.

**Changes made: **Figure 1 now presents the sequence of corpus selection, passage selection, coding, thematic grouping, ecocritical and Anthropocene interpretation, cross-text comparison, validation, and synthesis.

**Location in the revised manuscript: **Figure 1 and its accompanying explanatory paragraph in the Methodology section.

 2. Analysis

 Comment AN1

**Reviewer comment: **Minimize plot summary and replace it with close reading of scenes, images, metaphors, symbols, and plot structures.

**Response: **We agree.

**Changes made: **Descriptive plot material has been reduced, while the revised analysis focuses on the ocean, lifeboat, fear, Richard Parker, Snowman’s name, ruined landscapes, scarcity, engineered species, and the narrative contrast between spiritual survival and dystopian aftermath.

**Location in the revised manuscript: **Discussion throughout.

 Comment AN2

**Reviewer comment: **Ground theoretical concepts in textual evidence; for example, show how ‘slow violence’ manifests in Oryx and Crake.

**Response: **We agree and have strengthened the connection between theory and textual evidence.

**Changes made: **Slow violence is now linked to the novel’s accumulation of gradual ecological and social damage—resource depletion, normalized biotechnology, environmental deterioration, and the delayed consequences embodied in Snowman’s post-catastrophic existence. Dark ecology and trans-corporeality are likewise tied to specified textual situations.

**Location in the revised manuscript: **Discussion, analysis of Oryx and Crake’s ecological deterioration and Snowman’s embodied vulnerability.

 Comment AN3

**Reviewer comment: **Build a stronger synthesis showing what becomes visible only when the novels are read together.

**Response: **We agree and have made comparative synthesis more explicit.

**Changes made: **The revised discussion argues that reading the novels together reveals a shared anxiety about human dependence on nonhuman systems but divergent ecological ethics: Martel emphasizes adaptive coexistence and ethical formation, whereas Atwood emphasizes domination, technological overreach, and irreversible ecological loss.

**Location in the revised manuscript: **Comparative synthesis paragraphs in the Discussion and Findings sections.

 3. Literature Review

 Comment LR1

**Reviewer comment: **Delete citations not related to ecocriticism, including tourism, humanitarian assistance, and cartoons.

**Response: **We agree.

**Changes made: **These tangential citations have been removed from the literature-review argument, and unnecessary entries have been removed from the reference list.

**Location in the revised manuscript: **Literature Review and References.

 Comment LR2

**Reviewer comment: **Organize the Literature Review into three concise strands.

**Response: **We agree and have reorganized the review thematically.

**Changes made: **The three strands are: (1) ecological collapse, technological exploitation, and environmental ethics in Oryx and Crake; (2) spiritual ecology, survival, and human–nonhuman relations in Life of Pi; and (3) comparative ecocritical and Anthropocene scholarship establishing the unresolved research gap.

**Location in the revised manuscript: **Literature Review throughout.

 Comment LR3

**Reviewer comment: **Explain the relationship between postcolonial theory and ecocriticism if postcolonial theory remains in the framework.

**Response: **We agree.

**Changes made: **Postcolonialism is no longer treated as a separate analytical framework. Where postcolonial context remains relevant, it is explained as a contextual dimension of ecocriticism concerned with environmental inequality, human–nonhuman hierarchies, displacement, and uneven ecological vulnerability.

**Location in the revised manuscript: **Literature Review and Methodology, contextual discussion of postcolonial ecocriticism.

 4. Writing and Structure

 Comment WS1

**Reviewer comment: **Shorten the Introduction and focus on the research gap rather than plot summaries.

**Response: **We agree.

**Changes made: **The Introduction has been shortened and refocused on the ecological problem, the absence of a sustained comparison, the rationale, research questions, and the study’s intended contribution. Plot information has been retained only when needed to establish the comparative premise.

**Location in the revised manuscript: **Introduction throughout.

 Comment WS2

**Reviewer comment: **Ensure that the Discussion provides new information rather than repeating the Introduction and Literature Review.

**Response: **We agree.

**Changes made: **Repeated contextual material has been reduced. The Discussion now develops claims through textual interpretation and ends analytical units with cross-novel synthesis.

**Location in the revised manuscript: **Discussion throughout.

 Comment WS3

**Reviewer comment: **Break up long paragraphs into more digestible units.

**Response: **We agree.

**Changes made: **Long paragraphs have been divided according to claim, textual evidence, theoretical interpretation, and comparative implication. Transitional sentences have also been added to improve progression.

**Location in the revised manuscript: **Throughout the revised manuscript, especially the Literature Review and Discussion.

 5. Conclusion

 Comment C1

**Reviewer comment: **Narrow the Conclusion to the study’s specific contribution and avoid far-reaching claims about connecting literary theory and ethics worldwide.

**Response: **We agree and have narrowed the conclusion.

**Changes made: **The revised conclusion now emphasizes the specific insight produced by comparing the two ecological imaginaries: survival can be represented either as ethically formative coexistence or as the diminished remainder of anthropogenic ecological failure. Universal claims have been removed or qualified.

**Location in the revised manuscript: **Conclusion, contribution and implications paragraphs.

 6. References

 Comment R1

**Reviewer comment: **Delete unnecessary citations and citations not used in the essay.

**Response: **We agree.

**Changes made: **The references and in-text citations have been audited for relevance and correspondence. Unused or tangential entries and unnecessary citations have been removed, and page numbers have been supplied where required for direct quotations or closely attributed concepts.

**Location in the revised manuscript: **References and in-text citations throughout.

 Closing statement

 We again thank the reviewer for the constructive assessment. We believe that the revisions have produced a more focused, methodologically transparent, textually grounded, and comparatively integrated study. We hope that the revised version adequately addresses all concerns and recommendations.

 Sincerely,
